# Planning Non‐Linear Trajectories for No‐Touch Thermal Ablation Using Passive Steerable Needle With Controlled Stiffness and PSAM

**DOI:** 10.1049/htl2.70033

**Published:** 2025-11-19

**Authors:** Antoine Morin, Juan Manuel Verde, Lennart Rubbert, Alain Garcia, Caroline Essert

**Affiliations:** ^1^ ICube University of Strasbourg Strasbourg France; ^2^ IHU Strasbourg Strasbourg France; ^3^ INSA Strasbourg Strasbourg France

**Keywords:** decision support systems, needles, optimisation, surgery

## Abstract

Percutaneous thermal ablation using a passive steering needle has the potential to help reduce procedure risks and enable multiple ablations with a single insertion. However, manual planning of such procedures is challenging, time‐consuming, and typically done using CT‐scans or tools designed for rigid or active steering needles. We introduce the PSAM approach which is based on an enhanced version of the particle swarm optimization with a memory component. This tool calculates optimal trajectories for no‐touch thermal ablations, considering safety distances from obstacles such as bones and organs, and the amount of ablation affecting healthy tissue. Experiments were conducted on a passive steerable needle with controlled stiffness named ARC needle recently developed. The results are then compared with a semi‐exhaustive Monte‐Carlo approach and a manual planning by a trained surgeon using conventional rigid needles, and ranked by a second expert. The results demonstrate the interest and potential of the ARC needle and the PSAM approach to achieve the no‐touch approach and avoid multiple insertions.

## Graphical abstract

This study introduces PSAM, a planning method based on an enhanced particle swarm optimization with memory, to optimize trajectories for no‐touch thermal ablation using a passively steerable needle. It accounts for anatomical safety constraints and minimises damage to healthy tissue. Experiments with the new ARC needle show that PSAM outperforms manual and Monte Carlo planning, demonstrating its potential to reduce procedure risks and achieve effective ablations with a single insertion.

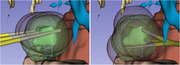

## 1 Introduction

Percutaneous thermal ablation (PTA) is a minimally invasive procedure that has become a first line treatment in certain patients, particularly for liver tumors where resection is not feasible due to tumor location, patient comorbidities, or insufficient liver reserve. Only a small percentage of liver tumor patients are eligible for surgical resection [1], making PTA an increasingly important option due to its reliability, safety, and effectiveness in local tumor control [2, 3]. Among the various thermal ablation technologies, radiofrequency ablation (RFA) and microwave ablation (MWA) are the most widely used due to their high clinical efficacy and favorable safety profiles [2, 3]. RFA uses high‐frequency alternating current to generate heat, while MWA employs electromagnetic waves to create larger and faster ablation volumes. Other techniques, such as cryoablation and high‐intensity focused ultrasound (HIFU), offer additional options based on tumor characteristics and institutional resources. Advancements in imaging guidance and ablation techniques continue to enhance the role of PTA in managing liver tumors and other malignancies.

**FIGURE 1:**
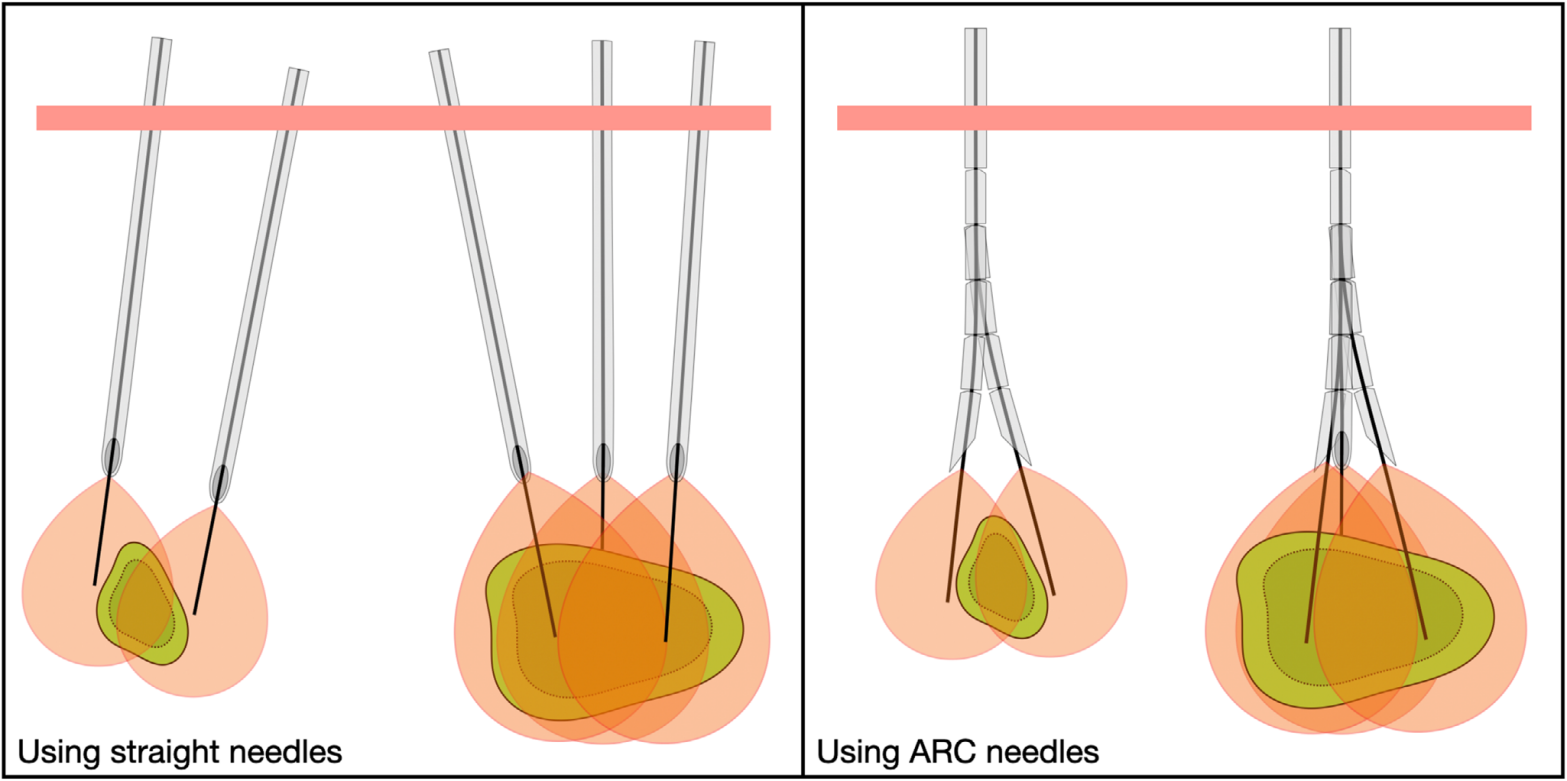
Illustration of thermal ablation on two tumor sizes and shapes: (left) using straight needles, and (right) using ARC needles. In all cases, the tumor (dark green) and its margin (light green) are surrounded by multiple ablation volumes (red), each created by an applicator (black line) inserted inside a hollow needle (gray) that doesn't enter the tumor. The needle is inserted through the skin (pink rectangle) either multiple times (straight needles, left) or only once (ARC needles, right).

One advanced technique gaining attention is the no/partial‐touch approach in PTA [4], which involves positioning the applicator around the tumor to deliver energy without directly penetrating it, only possibly entering the tumor with the catheter. This method aims to minimize the risk of tumor seeding and local recurrence by avoiding direct contact with the tumor, thereby creating a safety margin around it [5]. However, this approach is challenging as it usually requires multiple insertions of a straight needle to fully cover the tumor (Figure 1, left). This complexity increases the risk of unablated tumor persistence and early recurrence, as well as complications. It demands precise planning and imaging guidance to avoid critical structures and ensure complete ablation, necessitating advanced technical expertise and tools to ensure adequate thermal energy delivery while avoiding critical structures [6].

To address these challenges, various automatic or assisted planning tools have been proposed in the literature [7, 8]. These tools aim to simplify the planning process and improve the accuracy of needle placement. However, these approaches only address trajectories for straight or active steering needles involving multiple insertions. The introduction of passive‐steering beveled needles with high bending capabilities offers new opportunities and challenges [9, 10, 11]. These needles enable more flexible and precise targeting, allowing for non‐linear trajectories that can better navigate around critical structures and achieve effective no‐touch ablations. However, the planning process is even more complex [12]: manually planning a no‐touch ablation with non‐linear trajectories could take a trained surgeon up to 5 h due to trajectories being non‐linear, thus increasing the degrees of freedom. Therefore, a computer‐assisted planning tool is essential to optimize the use of passive‐steering beveled needles and fully realize the benefits of the no‐touch approach in PTA.

This paper introduces particle swarm with adaptive memory (PSAM), an enhancement of the particle swarm optimization (PSO) method, which we applied to automatic PTA planning for non‐linear trajectories, using the ARC needle previously presented in our recent work [13]. The approach was compared with a semi‐exhaustive Monte Carlo method and a manual planning executed by a trained surgeon on 15 cases of colorectal liver metastasis. A second trained surgeon semi‐blindly ranked the methods to assess the feasibility and relevance of the proposed solutions. The following sections detail the method, the experiment, and the results.

## 2 Methods

### 2.1 The Passive‐Steering Beveled Needle

**FIGURE 2:**
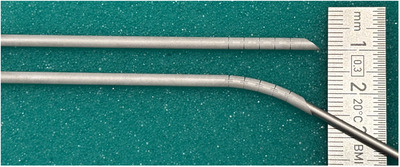
ARC needles. Top: locked joints in a straight, rigid state. Bottom: unlocked joints allowing passive bending (manually bent here).

The ARC needle, previously introduced briefly in our earlier work [13], is recalled here for clarity. The ARC needle features a straight, hollow main body and a multi‐articulated tip with a bevel. Its particularity is the ability to selectively lock or unlock one or more flexure joints in the articulated tip, allowing it to switch between a straight configuration and various predefined bending configurations [14]. This enables controlled passive steering of the tip when approaching the target, making it well‐suited for robotized telemanipulation applications. Our study employs an ARC needle of 2.8 mm diameter, with five flexure joints (Figure 2), each capable of bending up to 6 degrees. The flexure joints are axially spaced by 3.5 mm, with the beveled distal section extending to 5 mm. Our previous study was focused on the performance and behavior during freehand insertion in phantom gel of multiple designs of ARC needles. Considering the results obtained, the curvature provided by the chosen ARC needle is suitable for the automatic planning. In this study and experiment, we implement the observed performance of the ARC needle for automatic planning. This needle is used for no‐touch thermal ablations (Figure 1, right) where it is first inserted in a fully locked straight direction toward the tumor. At a designated flexion point during insertion, all flexure joints are unlocked, allowing the needle to deflect and avoid direct contact with the tumor. This process can be repeated around the tumor, by withdrawing the needle to the flexion point, then rotating it to the desired direction of bending, and reinserting it in a different curved direction. Each non‐linear trajectory thus shares the same flexion point. Following the peritumoral ablations, the surgeon may opt to perform one or two additional ablations (proximal and distal) within the tumor to ensure complete coverage. Thermal ablation is executed by deploying a catheter through the needle and applying microwave energy.

While in theory it is possible to unlock each flexure joint one by one for more refined trajectories, and to use different flexion points along the insertion depth, in this first study we have simplified the problem by considering that all flexure joints would be released at once at the same location before executing the final curved trajectory, leaving more complex scenarios for future works.

### 2.2 Needle Placement Constraints and Objectives

Similar to most conventional planning methods for PTA using straight needles, our approach involves a multi‐objective optimization. The primary goals are to ensure complete coverage of the targeted tumor and its margin, while preserving surrounding healthy tissues and avoiding critical structures. Following established guidelines [15, 16], we enforced a 10 mm safety margin around the tumor.

Several hard constraints have been defined: the needle must not intersect with any obstacles (such as bones or risky anatomical structures), the needle's flexure joints must remain within the liver when unlocked, and the catheter must be deployed inside the liver. We avoid insertion in the back of the patient, as the dataset's CT‐scans were captured with all patients lying on the back. Soft constraints include maintaining a security distance from any obstacle, minimizing the number of ablations required, and reducing the amount of healthy tissue affected.

To reflect these constraints, the automatic planning process optimizes each criterion of a score function defined by Equation (1). Here M is defined as the percentage of the total volume of the shape comprising the tumor and its margin that is effectively ablated, that we want to maximize. N is the number of ablations performed around the tumor, that we seek to minimize.

H corresponds to the percentage of destroyed healthy tissue (excluding the tumor margin) within the ablation volume. As the objective is to encompass both the tumor and a safety margin during ablation, a portion of surrounding healthy tissue will be unavoidably affected. The metric H is designed to quantify the trade‐off between maximizing pathological tissue coverage and minimizing collateral damage to healthy tissue.

D represents the normalized minimal distance between the planned trajectories and surrounding obstacles, as defined in Equation (2). Here, d is the smallest distance between a trajectory and an obstacle, obtained by computing the pointwise distances from the trajectory to each obstacle and subtracting the needle radius. Both trajectories and obstacles are sampled at intervals below 1 mm to generate these distances. In this formulation, D serves to penalize plans in which the needle approaches an obstacle more closely than a 5 mm safety margin, which should be respected as best as possible during trajectory planning.

Weights $w_{1}$, $w_{2}$, and $w_{3}$ can be chosen according to the final user's preferences. In our study, they were, respectively, set to 0.05, 0.2, and 0.5, to reflect the preferences of our surgeon. The weight distribution, along with the redundant information shared among M, H, and N, ensures that any modification in the planning is accurately reflected in the variation of the Score. This redundancy, combined with the strong penalization introduced by D, promotes a well‐balanced Score and helps maintain equilibrium across the evaluation criteria. Weights were chosen to ensure that a score close to 1 indicates that the ablations cover nearly 100% of the tumor and its margin while adhering to all soft constraints.

(1) $$Score=M-w_{1}N-w_{2}H-w_{3}D$$

(2) $$D=max\left(\frac{5-d}{5},0\right)$$

As the Score function only evaluates the intervention's planning, it does not reflect the surgeon's own performance during the intervention. An automatic planning with a high Score is therefore designed to reduce the risk of complications during or after the intervention by respecting the different constraints. The automatic planning process begins with the patient's segmentation as input. Automatic planning is defined by several variables: an entry point on the skin (selected from a pre‐processed list), the number of ablations (ranging from 1 to 8), the depth of the flexion point, and a rotational shift of the first angle within the range [0,2π/N]. Each method incorporates seeded randomness to ensure reproducibility. Automatic planning outputs a set of trajectories for a single needle, with a simulation of the thermal ablations. The goal of the automatic planning is to position ablations around the tumor, ensuring that the needle does not make direct contact with it.

### 2.3 PSAM: Memory‐Enhanced Particle Swarm Optimization for Non‐Linear Trajectory Planning

PSO [17] is a metaheuristic algorithm well‐suited for the modularity of passive‐steering beveled needles like the ARC needle. Its advantages include the ability to navigate a discrete search space without requiring gradient computation or differentiation of the optimization function. Additionally, PSO's high modularity allows for easy adaptation to the specific features of the needles used, such as the number of flexure joints, variable flexion point, and the number of flexure joints unlocked. However, PSO also has some limitations. In particular, PSO does not intrinsically address constrained optimization, and requires supplementary mechanisms such as penalty functions or repair strategies to enforce feasibility. In our context, anatomical constraints constitute a critical aspect of the problem, necessitating dedicated constraint‐handling approaches to avoid the algorithm getting lost in unfeasible spaces.

When adapting the original version of PSO to needle trajectories optimization, each particle in the swarm encodes a candidate configuration of trajectories. The position of the particle in the search space represents a specific set of parameters defining needle trajectories. At each iteration, particles update their positions based on their own inertia, their individual best‐known planning pbest (cognitive term) and the best planning discovered by the entire swarm gbest (social term). The two last terms influence the swarm toward high‐quality solutions while preserving exploration diversity.

At iteration t, for a parameter dimension i of a particle's planning configuration, the update of the internal search state (analogous to velocity), denoted $v_{i,t}$, follows Equation (3), constrained by bounds specific to that parameter. Here, w is the inertia weight moderating the influence of the previous update, $c_{1}$ and $c_{2}$ are the cognitive and social coefficients, and $\mu_{1}$, $\mu_{2}$ are uniformly sampled random variables in [0,1]. The terms $x_{i,\mathrm{pbest}}$ and $x_{i,\mathrm{gbest}}$ correspond to the best values found so far for dimension i by the particle itself and by the entire swarm, respectively. The planning configuration is then updated according to Equation (4), reflecting a new candidate solution in the search space.

(3) $$\begin{array}{ccc} v_{i,t} & = & w*v_{i,t-1}+c_{1}*\mu_{1}*\left(x_{i,pbest}-x_{i,t-1}\right)\\ & & +\;c_{2}*\mu_{2}*\left(x_{i,gbest}-x_{i,t-1}\right) \end{array}$$

(4) $$x_{i,t}=x_{i,t-1}+v_{i,t}$$

Zhang et al. [18] proposed a set of flexible parameters to choose from to adjust the convergence speed. Based on this paper, we chose w=0.724, $c_{1}=c_{2}=1.468$. These values ensure a good convergence speed, as well as a good balance between exploration (influenced by the cognitive coefficient $c_{1}$) and exploitation (influenced by the social coefficient $c_{2}$).

In our context, preprocessing can address a subset of hard constraints by reducing the search space and eliminating infeasible entry points. However, other hard constraints cannot be managed in this way and may prevent the algorithm from converging if not properly adapted. Hu et al. proposed a modification of PSO algorithm to solve constrained nonlinear optimization problems by only keeping track of solutions in the feasible space [19]. However, unfeasible particles kept being updated following Equations (3) and (4), leading in potential roaming in infeasible search space. Sun et al. introduced crossover operations between $x_{i,pbest}$ of different particles to generate additional candidate positions, even if infeasible [20, 21]. While effective, this requires repeated evaluations, which in our case would introduce prohibitive overhead given that planning is the computational bottleneck and the method is intended to run efficiently on a laptop. Coello Coello et al. proposed to use a corrective term, that replaces the gbest in Equation (3) with a previously visited feasible position randomly chosen [22]. The social term is discarded, each particle being guided instead by a different result in memory throughout the search, which can introduce bias in feasible regions, reduce diversity, and increase the risk of premature convergence. In a subsequent paper [23], they replace gbest with a ‘leader’, selected either as the global best among the feasible solutions (if any exist), or otherwise as the particle with the smallest constraint violation. While this ensures a stronger focus on feasibility, it biases the swarm toward constraint satisfaction throughout the optimization, which may come at the expense of exploration and objective improvement once feasibility is already achieved.

To preserve PSO's social exploration while having a controllable pull toward feasibility, we introduce particle swarm with adaptive memory (PSAM). This new variant of PSO stores encountered feasible solutions of both $x_{i,pbest}$ and $x_{i,gbest}$, in order to lead particles positioned in infeasible search space towards feasible search space, but this corrective term comes in addition to the social term instead of replacing it. Moreover, the corrective term is applied only when a particle is stuck in an infeasible region, which helps recover feasibility while preserving unbiased exploration elsewhere. PSAM updates the particles through three scenarios at each iteration:

- Particle defines a feasible solution and is updated using Equations (3) and (4).
- Particle's solution is infeasible and no feasible solution has been encountered, the particle is reinitialized with random values.
- Particle's solution is infeasible but feasible solutions exist in memory, Equation (3) is replaced by Equation (5), where $c_{1}=c_{2}=c_{3}$, $\mu_{3}$ is a random number in [0,1], and $x_{i,rHistory}$ is the position of a random feasible solution.

Scenario 2 uses randomness to find a feasible solution. For scenario 3 we implement a memory of known feasible solutions. This memory is used to guide the particle out of an infeasible search space.

(5) $$\begin{array}{cc} & v_{i,t}=w*v_{i,t-1}+c_{1}*\mu_{1}*\left(x_{i,pbest}-x_{i,t-1}\right)\\ & \;+\;c_{2}*\mu_{2}*\left(x_{i,gbest}-x_{i,t-1}\right)+c_{3}*\mu_{3}*\left(x_{i,rHistory}-x_{i,t-1}\right) \end{array}$$

As $x_{i,pbest}$ or $x_{i,gbest}$ can be undefined when infeasible, they default to the previous position of the particle $x_{i,t-1}$, canceling the corresponding term.

In the original PSO algorithm, the velocity of each dimension of a particle is updated using Equation (3), using the difference of position between a particle $x_{i,t}$, $x_{i,pbest}$ and $x_{i,gbest}$. In our search space, each dimension is bounded. Therefore, Equation (3) needs to be modified to update the velocity of a particle, as it is not appropriate to explore regions near the limits of a dimension. The difference between a particle $x_{i,t}$, and $x_{i,pbest}$ or $x_{i,gbest}$, that would be near the limit of a dimension, would not be big enough to make an important change in velocity. The particle position $x_{i}$ would then be stuck at the limit value of the dimension because of its own inertia. To solve this issue, we implemented bouncing bounds with Equation (6). By using this equation, when updating $x_{i,t}$, we ensure an appropriate update of the particle velocity $v_{i,t}$ near the limits of a dimension.

(6) $$\left\{\begin{array}{c} x_{i,t}=min_{i}+\left(min_{i}-x_{i,t}\right)\;if\;x_{i,t}<min_{i}\\ x_{i,t}=max_{i}-\left(x_{i,t}-max_{i}\right)\;if\;x_{i,t}>max_{i} \end{array}\right.$$

**FIGURE 3:**
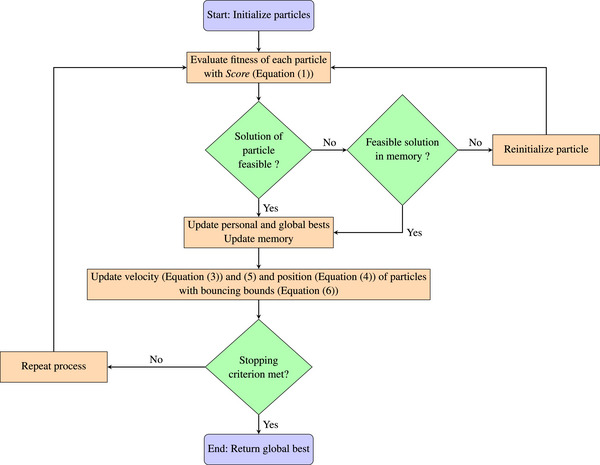
Diagram showing the algorithm of PSAM.

Figure 3 showcases the steps of our algorithm PSAM. To evaluate PSAM's performance, the algorithm deploys eight agents within the search space and terminates after 50 iterations without improvement.

### 2.4 Semi‐Exhaustive Approach Using Monte‐Carlo Sampling

A finite set of possible plannings exists for each tumor. Monte Carlo (MC) method [24] is used to randomly generate a sample of 1000 automatic plannings for each tumor, and explore the space of feasible needle trajectory configurations in a stochastic yet controlled manner. A score is associated to each planning using Equation (1). The highest score obtained for each tumor with this method is used as a benchmark score to evaluate PSAM method. The maximum number of ablations was bounded to 4 if a theoretical ablation volume could cover the margin, to reduce the possible randomness.

## 3 Experimental Validation

### 3.1 Dataset and Preprocessing

Our experiments were conducted on a subset of 15 portal venous phase contrast‐enchanced computed tomography (CT) scans from the publicly available colorectal‐liver‐metastases (CRLM) dataset, which provides preoperative imaging and the segmentations of the liver, tumors, and vessels of patients undergoing resection of colorectal liver metastases [25, 26]. The dataset, hosted by The Cancer Imaging Archive, consists of 197 subjects. A preliminary random selection of 25 cases was performed, from which the first 15 cases meeting ablatability criteria for ablation procedures as per [27] were retained.

For anatomical segmentation, the TotalSegmentator [28] module was used within 3D Slicer (version 5.8.0). This process generated volumetric segmentations of relevant anatomical structures. Furthermore, the 10 mm margin was created by expanding the tumor shape. Upon completion, each case was stored as a 3D Slicer scene, encapsulating both the segmented volumes and the associated annotations. This dataset was subsequently used as input for the automatic trajectory planning algorithms.

**FIGURE 4:**
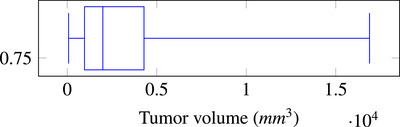
Tumor volume distribution in the dataset.

A list of possible entry points on the skin mesh was generated by evaluating each vertex to ensure that it met the following criteria: the needle path from the entry point to the tumor must be obstacle‐free, the operating table must not impede needle insertion, and the insertion angle relative to the organ (in this case, the liver) must not exceed 30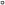. Figure 4 shows the volume distribution of the different tumors of the 15 cases selected. While most of the tumors are between $900\;{\text{mm}}^{3}$ and $4300\;{\text{mm}}^{3}$, selected cases also include small tumors ($75\;{\text{mm}}^{3}$) and large tumors ($16\;900\;{\text{mm}}^{3}$).

### 3.2 Description of the Experiment and Metrics

In this study, we focused on microwave ablation (MWA) procedures. Given the absence of comparable methods in the literature, we applied three distinct needle trajectory planning strategies to our dataset for comparative analysis. For each method, we used microwave ablation of 100 W performed during 10 min to ensure fair comparison between each planning. The first method, manual planning, reflects the current clinical practice. This approach involves a clinician determining the insertion points for multiple rigid needles, each intended to perform a single thermal ablation, to collectively achieve the desired treatment. A trained operator conducted this planning in two phases: initially, linear trajectories for the needles were plotted using the patients' CT scans; subsequently, a computer‐assisted tool (Ablation Planner^1^) calculated the ablation volumes based on these trajectories to assess the expected margins. Notably, the surgeon did not adjust the initial plans after utilizing this tool. Even without further adjustments, each manual planning took around an hour in average.

Then, the two algorithms PSAM and MC, respectively, described in Sections 2.3 and 2.4, were used to generate automatic plannings for ARC needles. Here the result of a planning is a single straight trajectory, ending with multiple curved trajectories for the articulated tip around the tumor. For both approaches, four configurations were tested: surrounding needles only (PSAM and MC), with an additional proximal ablation (“+Prox”), with an additional distal ablation (“+Dist”), and with both a proximal and distal ablations (“+Both”).

To evaluate each planning, we measured the number of insertions and ablations, the percentage of tumor covered (with and without margin), the efficiency of the planning (the percentage of the ablation covering tumor tissue), the minimum distance from any needle or catheter to any obstacle (excluding bones), and the execution time. Additionally, a second trained surgeon assessed the feasibility of all plannings and performed a semi‐blind ranking of the proposed solutions. While the manual planning, involving multiple insertions of straight needles, could not be concealed, the other two solutions were randomly assigned numbers to facilitate a blind ranking.

## 4 Results and Discussion

**FIGURE 5:**
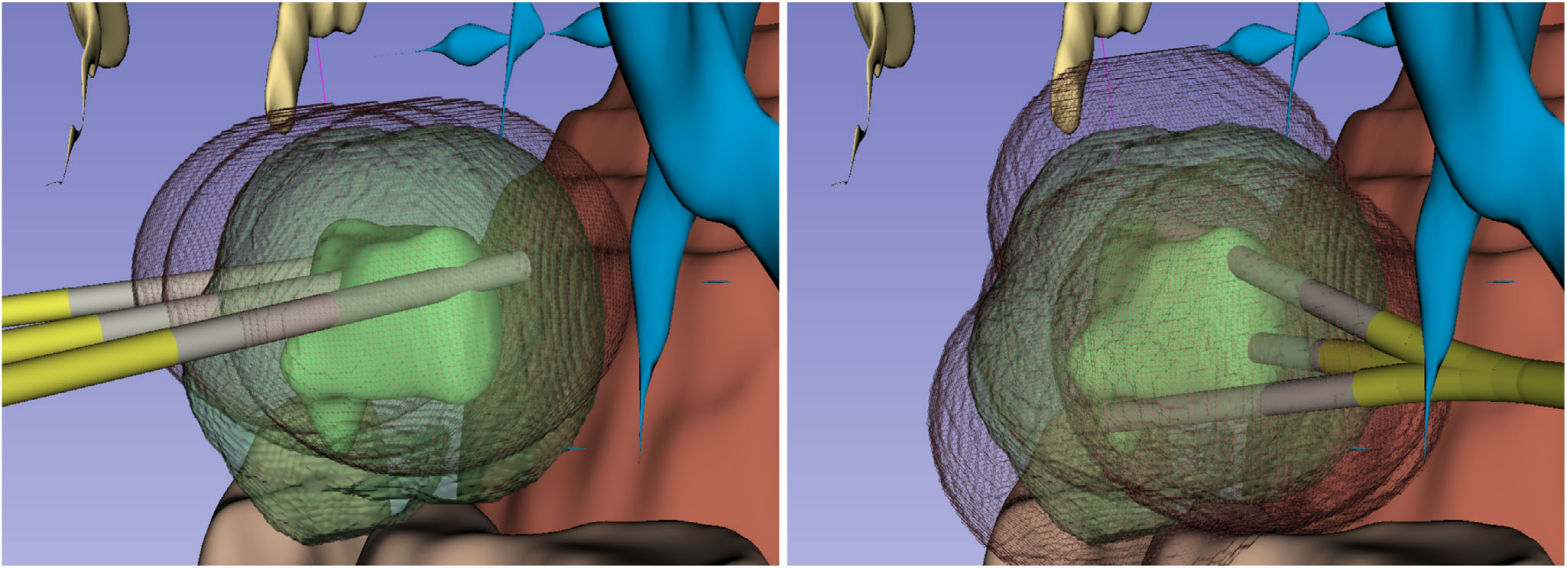
Comparison of manual (left) and PSAM (right) trajectory planning. The ARC needle is shown in yellow, the ablation applicator in gray, the tumor and its margin in green and semi‐transparent, and the estimated ablation volumes are displayed as dark wireframe meshes. Both are seen from the same viewpoint and angle of view in the 3D scene.

**FIGURE 6:**
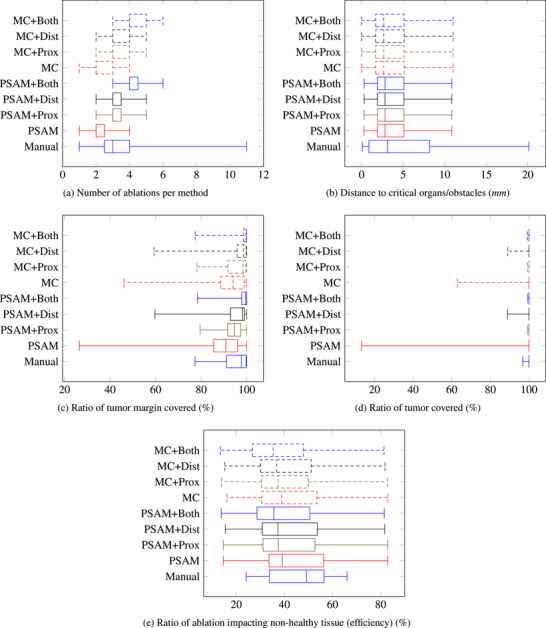
Results of manual and automatic planning approaches on 15 cases for five different metrics: number of ablations per method, distance to critical organs/obstacles, ratio of tumor margin covered, ratio of tumor covered, and ratio of ablation impacting non‐healthy tissue (efficiency).

The automatic plannings were run on a 13th Gen Intel(R) Core(TM) i9‐13900 CPU with 32 cores and 32GB of RAM. In the 15 cases chosen in this dataset, automatic planning approaches successfully found trajectories for each case. Table 1 summarizes the results obtained with manual planning and the two automatic planning approaches and Figure 6 shows the statistical distribution of the key metrics obtained. Figure 5 illustrates a result of the manual and automatic planning for the same case. The tumor and its margin are covered by the ablations performed by the catheter that is brought through the needles. Automatic planning also showcases the proximal and distal ablations going through the tumor. Note that both image show the 3D scene from the same viewpoint and angle of view, but the manual and automatic planning chose different insertion angles adapted to their respective particularities.

**TABLE 1:** Global results of manual and automatic planning approaches on 15 cases.

Method	Distance		Avg. coverage		Avg. efficiency	Ablations
	Avg.	Min.	Margin	Tumor	Efficiency	Avg. number
	[mm]	[mm]	[%]	[%]	[%]	
Manual	5.46 (±5.8)	0.09	94.6 (±7)	99.7 (±1)	46 (±14)	3.7 (±2.3)
PSAM	3.65 (±2.8)	0.31	84.8 (±19)	93.3 (±22)	44 (±19)	2.3 (±0.7)
PSAM +Prox	3.65 (±2.8)	0.31	93.1 (±6)	99.9 (±0)	43 (±18)	3.3 (±0.7)
PSAM +Dist	3.65 (±2.8)	0.31	93.7 (±11)	99.1 (±3)	43 (±18)	3.3 (±0.7)
PSAM +Both	3.65 (±2.8)	0.31	96.9 (±6)	100.0 (±0)	40 (±18)	4.3 (±0.7)
MC	3.79 (±3.3)	0	88.5 (±16)	96.7 (±10)	44 (±19)	2.5 (±0.7)
MC+Prox	3.79 (±3.3)	0	94.8 (±7)	99.9 (±0)	42 (±19)	3.5 (±0.7)
MC+Dist	3.79 (±3.3)	0	94.2 (±11)	99.2 (±3)	42 (±18)	3.5 (±0.7)
MC+Both	3.79 (±3.3)	0	97.2 (±6)	99.9 (±0)	40 (±18)	4.5 (±0.7)

In terms of coverage and efficiency, manual planning using multiple insertions of straight needles reached very high results. All algorithm‐generated solutions also achieved comparable results with only a single‐insertion using the no‐touch approach, highlighting the interest of performing this approach with the ARC needle. Manual planning maintained a slightly higher distance to risky structures, indicating marginally safer trajectories, though automatic trajectories remained within acceptable ranges according to the expert. We can however note that the standard deviation was lower for the automatic plannings, especially PSAM, indicating less variability in safety, as shown in Figure 6. Proximal and distal ablations in algorithm‐generated results enhanced tumor coverage and ensured complete ablation, with both PSAM +Prox and MC+Prox even slightly outperforming manual planning with fewer ablations on average. Statistical analysis comparing manual planning with PSAM automatic planning (alone, +Prox, +Dist, +Both) reports no statistically significant difference in the measured metrics (*p*‐values > 0.05). Only the number of insertions (for every variation of PSAM) and the number of ablations (only for PSAM alone) were different between both planning methods (*p*‐values < 0.05). These results highlight that combining the automatic planning tool with the use of ARC needles enables interventions on par with manual planning.

The expert deemed optimal automatic plannings feasible in 11 out of 15 cases. In the remaining 4 cases, solutions were found but not considered feasible in the final assessment, suggesting either a missing constraint, a missing anatomical structure in the segmentation, or a limitation due to the fixed maximal curvature imposed on each trajectory. Indeed, by activating all flexure joints, we favored easier freehand reproducibility at the expense of trajectory versatility. In these few cases, this sometimes resulted in tumors being too narrow for the imposed curvature, with excess damage to surrounding tissue and parts of the tumor or its margin insufficiently covered (see variability in Figures 6c and 6d). Such cases could be mitigated in future works by modulating the number of flexure joints released for each trajectory.

Figure 6e shows the impact of the ablation on healthy tissue can be reduced compared to manual planning with rigid needles. In the 11 feasible cases, the expert ranked manual planning last in 9 cases, highlighting the advantages of non‐linear trajectories and the no‐touch method over straight needles due to reduced risks when performing fewer insertions. When blindly ranking PSAM and MC, PSAM was preferred in 7 out of 11 cases, MC in 3, and manual planning with the multiple straight trajectories in only 1 case, indicating an advantage for PSAM. Furthermore, PSAM and MC proposed solutions averaging 2.4 and 2.5 ablations, respectively, with a single insertion, while manual planning averaged 3.7 ablations and insertions. Adding an additional ablation (proximal or distal) kept the average number of ablations below that of straight needles. One case included a tumor of 80 ${\mathrm{cm}}^{3}$ including the margin. For equal ablation performances, manual planning required 6 insertions and ablations while PSAM proposed a planning with only one insertion and 4 ablations.

Regarding the performance, the PSAM algorithm reached a convergence after 63 iterations and about 52 min of processing time on average. Even though the manual planning of thermal ablation using rigid needles takes about 40 min to 1 h, manual planning using passive steering needles can take up to 5 h.

In this study, we simplified the optimization by restricting bending to a full release of all flexure joints. Future work could explore varying the number of unlocked flexure joints for each needle independently, allowing for different curvatures and more versatile ablation configurations. Extensions of this approach could also allow to explore new needle designs by treating specifications as variables, helping to identify optimal designs for most ablations. Considering the performance of the automatic planning, next step would be to perform ex‐vivo experiments using both the ARC needle and the PSAM automatic planning.

## 5 Conclusion

In this paper, we explored automatic planning of percutaneous no‐touch thermal ablation using passive steering beveled needles with controllable stiffness, such as the ARC needle. Passive steering needles have the potential to perform multiple ablations with a single insertion, without putting as much constraints as active steering needle on the tissue. We proposed PSAM, an algorithm based on Particle Swarm Optimization capable of handling hard constraints and bounded dimensions. Automatic planning achieves outcomes comparable to manual planning while requiring fewer ablations and a single insertion. This confers multiple advantages: reduced invasiveness and risk, shorter procedure time, and facilitated access for no‐touch or partial‐touch techniques that improve tumor recurrence prevention, all without compromising safety or efficacy relative to manual planning with conventional straight needles. Such advantages were confirmed by a semi‐blind evaluation, where experts expressed a preference for automatic planning. Future work may focus on expanding the degrees of freedom to enhance versatility and clinical applicability.

## Author Contributions

**Antoine Morin**: conceptualization, data curation, formal analysis, software, methodology, visualization, validation, writing – original draft, writing – review and editing. **Juan Manuel Verde**: conceptualization, data curation, resources, supervision, writing – original draft, writing – review and editing, validation. **Lennart Rubbert**: conceptualization, resources, supervision, writing – original draft, writing – review and editing. **Alain Garcia**: validation. **Caroline Essert**: conceptualization, data curation, funding acquisition, methodology, project administration, resources, supervision, validation, writing – original draft, writing – review and editing.

## Funding

This work of the Interdisciplinary Thematic Institute HealthTech, as part of the ITI 2021–2028 program of the University of Strasbourg, CNRS and Inserm, was supported by IdEx Unistra (ANR‐10‐IDEX‐0002) and SFRI (STRAT'US project, ANR‐20‐SFRI‐0012) within the framework of France 2030. It has been partially funded under the framework of the French Investments for the Future Program, by French state funds managed within the “Plan Investissements d'Avenir”, by the ANR (reference ANR‐10‐IAHU‐02). It has also been partially funded by Région Grand Est under the SRESRI program.

## Conflicts of Interest

The authors declare no conflicts of interest.

## Data Availability

The data that support the findings of this study are available from the corresponding author upon reasonable request.

## Endnotes

1
https://github.com/naterex23/SlicerAblationPlanner
